# The prognostic value of Her4 receptor isoform expression in triple-negative and Her2 positive breast cancer patients

**DOI:** 10.1186/1471-2407-13-437

**Published:** 2013-09-24

**Authors:** Anna Machleidt, Stefan Buchholz, Simone Diermeier-Daucher, Florian Zeman, Olaf Ortmann, Gero Brockhoff

**Affiliations:** 1 Department of Gynecology and Obstetrics, University Medical Center, Caritas Hospital St. Josef, University of Regensburg, Landshuter Strasse 65, 93053 Regensburg, Germany; 2 Center for Clinical Studies, University of Regensburg, Regensburg, Germany

**Keywords:** Her4 expression, Her4 isoforms, qPCR, Triple-negative breast cancer, Her2 positive breast cancer

## Abstract

**Background:**

Not only four but rather seven different human epidermal growth factor receptor related (Her) receptor tyrosine kinases (RTKs) have been described to be expressed in a variety of normal and neoplastic tissues: Her1, Her2, Her3, and additionally four Her4 isoforms have been identified. A differential expression of Her4 isoforms does not, however, play any role in either the molecular diagnostics or treatment decision for breast cancer patients. The prognostic and predictive impact of Her4 expression in breast cancer is basically unclear.

**Methods:**

We quantified the Her4 variants JM-a/CYT1, JM-a/CYT2, JM-b/CYT1, and JM-b/CYT2 by isoform-specific polymerase chain reaction (qPCR) in (i) triple-negative, (ii) Her2 positive breast cancer tissues and (iii) in benign breast tissues.

**Results:**

In all three tissue collectives we never found the JM-b/CYT1 or the JM-b/CYT2 isoform expressed. In contrast, the two JM-a/CYT1 and JM-a/CYT2 isoforms were always simultaneously expressed but at different ratios. We identified a positive prognostic impact on overall survival (OS) in triple-negative and event-free survival (EFS) in Her2 positive patients. This finding is independent of the absolute JM-a/CYT1 to JM-a/CYT2 expression ratio. In Her2 positive patients, Her4 expression only has a favorable effect in estrogen-receptor (ER)-positive but not in ER-negative individuals.

**Conclusion:**

In summary, JM-a/CYT1 and JM-a/CYT2 but not JM-b isoforms of the Her4 receptor are simultaneously expressed in both triple-negative and Her2 positive breast cancer tissues. Although different expression ratios of the two JM-a isoforms did not reveal any additional information, Her4 expression basically indicates a prolonged EFS and OFS. An extended expression analysis that takes all Her receptor homologs, including the Her4 isoforms, into account might render more precisely the molecular diagnostics required for the development of optimized targeted therapies.

## Background

The Her (human epidermal growth factor related) receptor tyrosine kinases (RTK) comprise four homologous proteins (Her1-4), which are differentially expressed during development and functional maintenance of the normal mammary gland [1-4]. Spatiotemporally regulated RTK (co-)expression, however, is commonly disturbed in neoplastic mammary epithelium. 15% - 25% of breast cancers show Her2 receptor overexpression, which has a negative prognostic impact on the outcome of disease [5]. Specific Her2 receptor targeting with antibodies (e.g. trastuzumab and/or pertuzumab) or small molecule kinase inhibitors (e.g. lapatinib), usually applied in combination with chemotherapy or antihormonal therapeutic intervention, potentially prolongs the time to tumor progression and/or the overall survival rate of palliatively (metastatic) or (neo-)adjuvantly treated breast cancer patients [6]. Individual responsiveness, however, (based on Her2 overexpression/*her2* gene amplification) cannot be predicted, varies significantly, and spans from *de-novo* to acquired resistance to moderate and high susceptibility [7].

Her1 and Her3 receptor expression in breast cancer has been described to be associated with a poor course and outcome of disease [8,9]. In contrast, the prognostic (and predictive) value of Her4 receptor expression is uncertain [10-16]. Both a positive and a negative impact of Her4 (co-)expression has been reported. This inconsistency can be conceivably attributed to the complex Her4 signaling capabilities, which among other reasons, might result from the differential expression of alternatively spliced Her4 isoforms [17,18]. In fact, at least four different Her4 variants (JM-a/CYT1, JM-a/CYT2, JM-b/CYT1, and JM-b/CYT2) can be generated by differential Her4 mRNA splicing. The juxtamembrane domain JM-a, but not JM-b, contains a cleavage site for the tumor-necrosis-factor-α-converting enzyme (TACE). CYT1/CYT2 intracellular domains have been demonstrated to differentially trigger intracellular signaling upon further Her4 activation by γ-secretase [19,20]. Hence, the Her4 types differ in both function and signaling capabilities. Overall, not only four different Her receptors (Her1-4) but rather seven homologs (Her1-3 plus four Her4 isoforms) can potentially be coexpressed [17]. The prognostic value of isoform-related Her4 expression in breast cancer is, however, unknown.

The aim of this study was to evaluate the prognostic impact of Her4 isoform expression in well-characterized subgroups of breast cancer patients. Therefore, we analyzed the differential expression in primary tumor tissues of so-called triple-negative breast cancer (TNBC, i.e. estrogen, progesteron and Her2 receptor-negative) and Her2 positive patients by quantitative real-time polymerase chain reaction (qPCR). Isoform-specific Her4 expression was correlated with the outcome of disease in terms of event-free and overall survival. Extensive statistical analysis was applied to evaluate the prognostic value of Her4 (isoform) expression in well-defined TNBC and Her2 positive breast cancer cohorts.

## Methods

### TNBC and Her2 positive breast tumor samples

The patients were diagnosed between 1992 and 2008. Basic patient characteristics are summarized in Table 1.

**Table 1:** Basic TNBC and Her2 positive patient characteristics

	**TNBC**	**Her2 positive**
# Total	76 (100%)	96 (100%)
Median patient age	54.3 y (range 28 – 83)	54.0 (range 24 – 79)
# Grading 1	1 (1.3%)	0 (0%)
# Grading 2	20 (26.3%)	39 (40.6%)
# Grading 3	54 (71.1%)	56 (58.3%)
# Grading unknown	1 (1.3%)	1 (1.0%)
# Stage 1	17 (22.4%)	17 (17.7%)
# Stage 2	41 (53.9%)	42 (43.8%)
# Stage 3	9 (11.8%)	22 (22.9%)
# Stage 4	4 (5.3%)	13 (13.5%)
# Staging unknown	5 (6.6%)	2 (2.1%)
pNO (initial diagnosis)	41 (53.9%)	33 (34.3%)
pN+	29 (38.2%)	58 (60.4%)
pNX	6 (7.9%)	5 (5.2%)
Metastatic patients (initial diagnosis)	14 (18.4%)	13 (13.5%)
Median OS [months]	55.8 (range 0.9 – 238)	41.2 (range 13.0 – 193.5)
Median EFS [months]	50.9 (range 0.9 – 197.9)	33.3 (range 7.8 – 114)

### Breast tumor samples and patient characteristics of TNBC

Cryo-preserved tissues (n = 24), as well as formalin-fixed and paraffin-embedded tissue blocks (n = 52) from 76 female patients with triple-negative breast cancer derived from the archive of the Institute of Pathology (University of Regensburg, Germany) were included in the study. Clinical data were acquired by the Tumor Center e. V, Regensburg.

The median patient age at diagnosis was 54.3 years, with a range of 28 to 83 years. A major portion of patients were diagnosed between 60 and 69.9 years of age. Another peak of incidence, as is typical for triple-negative breast cancer, was found in a younger patient age group i.e. individuals between the ages 40 and 54 years. 97.4% of patients underwent surgery, 61.8% of them had breast-conserving surgery, 35.5% underwent a mastectomy. 75.0% of patients were treated with chemotherapy. 55.3% of patients received one chemotherapy regimen, 13.2% had two and 6.6% had three or more chemotherapy regimes. 8 patients received chemotherapy in a neoadjuvant setting. Chemotherapeutic regimes were mainly Taxane- and Antraycline-based. Two patients were treated with aromatase inhibitor (Anastrozol) having a hormone receptor-positive second breast carcinoma. 35.1% of the patients died and 44.6% suffered from a recurrence of breast cancer. 4 patients showed metastasis at the time of primary diagnosis.

### Breast tumor samples and patient characteristics of Her2 positive patients

Tissues from 96 female patients were examined regarding their expression of Her4 receptor splice variants. We included 26 (27.1%) cryo-preserved and 70 (72.9%) paraffin-embedded specimens. 91 of the 96 patients (94.8%) underwent surgery as primary therapy, 50 patients (52.1%) received breast-conserving surgery, and 26 patients (27.1%) had a mastectomy. In 20.9% the type of operational therapy was unknown (n = 20). 80 (83.3%) patients underwent an adjuvant chemotherapy regimen, 9 patients (6%) received neoadjuvant chemotherapy. 79 patients (82.3%) were treated with trastuzumab. 58 out of them (60.4%) received trastuzumb at primary diagnosis, 17 (17.7%) received trastuzumab upon recurrence of disease and 4 patients (4%) were treated with trastuzumab both times. 13 patients (13.5%) had metastasis at the time of primary diagnosis.

### Control tissue samples

Benign mammary tissue samples (total n = 35, cryo-preserved n = 13, paraffin-embedded n = 22) were included in the study to compare Her4 expression in tumor tissues to Her4 expression in non-malignant tissues. This non-malignant material was identified by a pathologist and derived from a non-tumorous and separately localized region of tumor patients’ tissue samples.

### RNA isolation, cDNA synthesis and real-time qPCR

RNA isolation of cryo-preserved tissues was performed using Trizol (peqGOLD TriFast), 70% Isopropanol and RNeasy Mini Kit (Qiagen, Hilden, Germany) according to the manufacturer’s protocol. RNA samples were treated with 10 μl DNase I (Roche Diagnostics, Mannheim, Germany) to eliminate potential DNA contamination.

The miRNeasy RNA Isolation Kit (Qiagen) was used to extract RNA from paraffin-embedded tissues. For synthesis of cDNA a template of 0.5 μg total RNA was used. According to the manufacturer’s instructions (Transcriptor First Strand cDNA Synthesis Kit/Roche), the reaction contains random hexamers (Promega, Mannheim, Germany), reverse transcriptase (Promega), dNTP-mixture and RNAse inhibitor. To identify false-positive amplification due to contamination of chromosomal DNA, the reactions were performed in duplicate in the presence and absence of reverse transcriptase.

Probes and primers (Metabion, Martinsried, Germany) for Her4 isoform-specific real-time PCR were synthesized based on the PCR design published by Junttila et al. [21], (Table 2). The original approach, which was performed using the Taq-man technology, was transferred to the Light Cycler (LC) 480 platform (Roche Diagnostics, Mannheim, Germany). The transfer was established and validated by e.g. optimizing amplification efficiencies and verifying amplification specificities.

**Table 2:** Her4 isoform-specific primers and probes

		
JM-a	Forward	5′-CCA CCC ATC CCA TCC AAA-3′
	Reverse	5′-CCA ATT ACT CCA GCT GCA ATC A-3′
	Probe	5′-Fam-ATG GAC GGG CAA TTC CAC TTT ACC A-Dabcyl-3′
JM-b	Forward	5′-CCA CCC ATC CCA TCC AAA-3′
	Reverse	5′-CCA ATT ACT CCA GCT GCA ATC A-3′
	Probe	5′-Fam-CTC AAG TAT TGA AGA CTG CAT CGG CCT GAT-Dabcyl-3′
CYT1	Forward	5′-CAA CAT CCC ACC TCC CAT CTA TAC-3′
	Reverse	5′-ACA CTC CTT GTT CAG CAG CAA A-3′
	Probe	5′-Fam-TGA AAT TGG ACA CAG CCC TCC TCC TG-Dacyl-3′
CYT2	Forward	5′-CAA CAT CCC ACC TCC CAT CTA TAC-3′
	Reverse	5′-ACA CTC CTT GTT CAG CAG CAA A-3′
	Probe	5′-Fam-AAT TGA CTC GAA TAG GAA CCA GTT TGT ATA CCG AGA T-Dabcyl-3

Real-time PCR was performed using fluorescent oligonucleotid LC480 hybridization probes (Metabion). A calibration standard as well as probes and primers annealing to mRNA of β-actin were used as internal reference and for comparison of successive experiments. Three different β-actins were used (Table 3) matched to the length of the splice variants, for an exact comparability between target and control in both paraffin-embedded and cryo-preserved tissues.

**Table 3:** β-actin primers and hybridization probes (Metabion)

		
β-actin probe (LC Red)		
5′-LCRed-610-TGA CCC AGA TCA TGT TTG AGA CCT TCA ACA C-BHQ-2-3′		
β-actin	Forward1	5′-GGA GCA CCC CGT GCT GC-3′
	Reverse1	5′-GCG TAC AGG GAT AGC ACA GCC-3′
β-actin	Forward2	5′-CCT GAA CCC CAA GGC CAA CC-3′
	Reverse2	5′-GTG GTA CGG CCA GAG GCG-3′
	Forward3	5′-ATC TGG CAC CAC ACC TTC TAC AAT-3′
	Reverse3	5′-CCG TCA CCG GAG TCC ATC A-3′

A calibration standard comprised of a mixture of paraffin-embedded cell lines (ZR.75.1, MCF-7, T47D) expressing the splice variants served as a second internal control. Every sample was carried out in triplicate.

PCR was carried out in a final volume of 10 μl containing 2.5 μl cDNA template (1:5 attenuation), 5 μl LC480 Probes Master (Roche), 1 μl probe and 1.5 μl primers (0.75 μl primer β-actin, 0.75 μl primer target). Probes were labeled with fluorescent reporter dyes FAM (Her4 isoform probes) or LC Red (β-actin probes). Thermal cycling started with the pre-incubation at 95°C for 10 minutes. Then amplification was carried out for 45 cycles, initiated with 30 s at 60°C followed by 15 s at 95°C on a LC480.

For unifying qPCR results derived from the analysis of cryo-preserved and paraffin-embedded tissues, we introduced a conversion (normalization) factor that took into account different amplification efficiencies. The factor was generated by analyzing matched paraffin-embedded/cryo-preserved tissue samples of the same patient (n = 26). This systematic comparison revealed a 4.9-fold higher amplification efficiency of RNA derived from frozen tissues.

### Ethical approval

All experiments were approved by the Ethics Committee of the University of Regensburg (permission no.: 13-101-0012). All patients included in the experiments provided written informed consent based on a procedure approved by the Ethics Committee of the University of Regensburg (permission no.: 05–176). Overall, all experiments were performed in accordance with relevant institutional and national guidelines, regulations and approvals.

### Statistical analysis

Categorical data are presented as frequency counts and percentages, continuous variables as median and range. To compare Her4 expression levels between different groups, the non-parametric Mann–Whitney U Test was used. To analyze the correlation between Her4 isoforms and clinicopathologic parameters, Spearman’s rank correlation coefficients were calculated.

Event-free survival (EFS) and overall survival (OS) times were calculated from the date of diagnosis to the date of event (tumor recurrence or death), respectively. Patients without an event were classified as censored at the last date to be known event free and alive. To assess the prognostic value of Her4 (JM-a) expression on EFS and OS, univariable and multivariable Cox proportional hazard models were calculated. Variables with p < 0.10 in a univariable analysis were entered into a multivariable model. Hazard ratios (HR) and corresponding 95% confidence intervals (CI) were calculated according to the likelihood ratio test, and a two-sided P value of < 0.05 was considered to indicate statistical significance. All analyses were performed using IBM SPSS Statistics 20.0 and SAS 9.3 (SAS Institute Inc., Cary, NC, USA).

## Results

We performed a Her4 isoform-specific expression analysis in 76 TNBC and 96 Her2 positive tissues of female tumor patients. If available, the associated non-malignant tissues were examined in addition (matched pair analysis, n = 26).

### Her4 isoform expression in TNBC and Her2 positive patients

**Figure 1:**
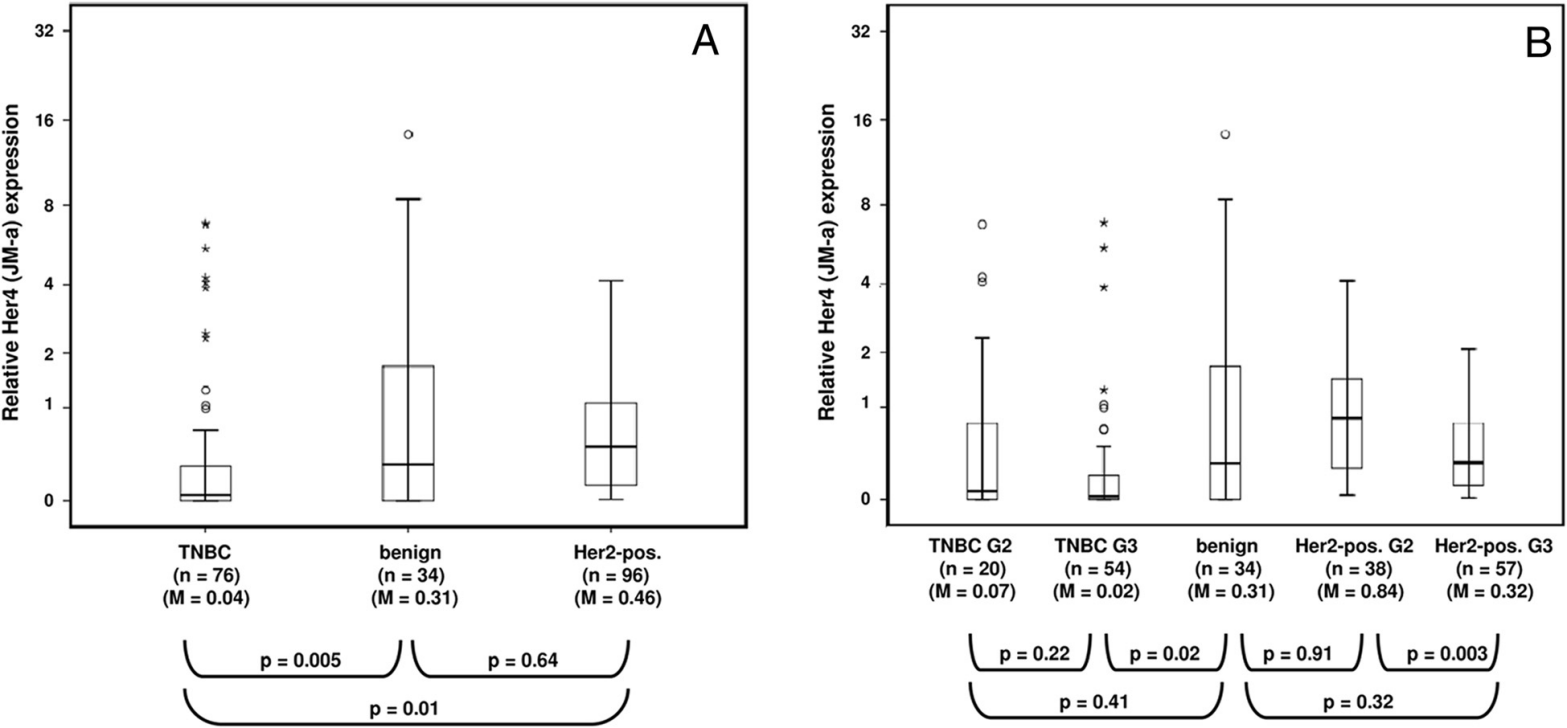
**Box Plot diagram showing relative Her4 (JM-a) expression in TNBC, benign tissues, and Her2 positive breast cancer tissues irrespective of grading (A) and differentiated in terms of grading 2 and grading 3 (B), respectively”.** Numbers of specimens analyzed (n) and median expression levels (M) are indicated”. P-values indicate expression levels between compared groups (Mann–Whitney U test). Note the log-2 based data displayed on the y-axes.

We found the Her4 juxtamembrane JM-a splice variants expressed at a frequency of 18.4% (14 of 76) in triple-negative and 43% (41 of 96) in Her2 positive breast cancer samples. The relative expression level of Her4 (JM-a) differs up to 6.9-fold in TNBC tissues and up to 4.1-fold in Her2 positive tissues (Figure 1A).

JM-b receptor variants were not found in any of the examined breast tissues. JM-a/CYT1 and JM-a/CYT2 isotypes were always simultaneously expressed, however CYT1/CYT2 expression ratios vary and range from 0.12 to 11 in TNBC specimens and from 0.38 to 3.77 in Her2 positive tissues.

### Her4 (JM-a) expression in non-malignant (control) tissues

**Figure 2:**
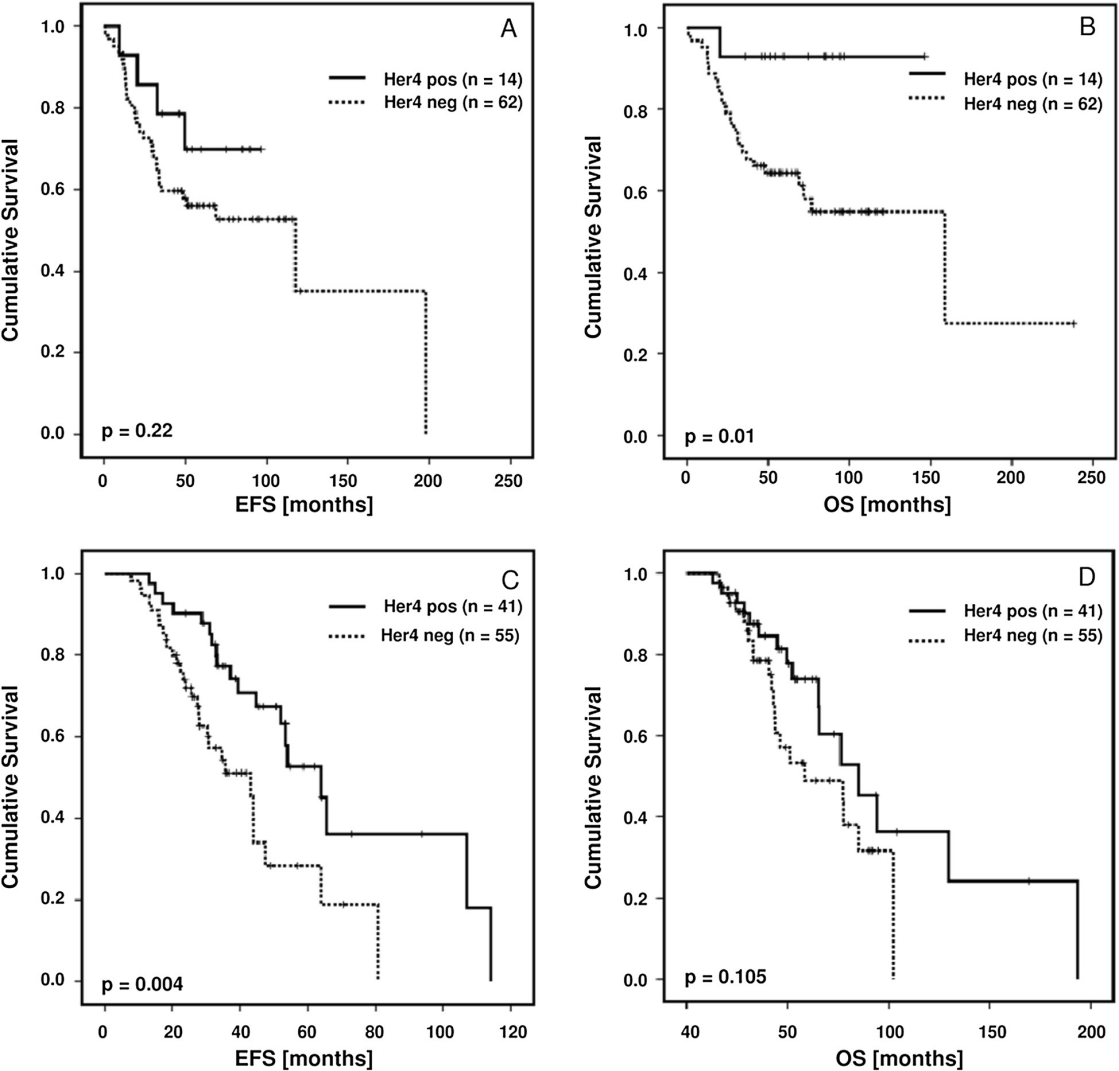
Kaplan-Meier curves of the effect of Her4 (JM-a) expression on EFS (A) and OS (B) of TNBC (A and B) and Her2 positive patients (C and D), respectively.

Figure 1A: The relative Her4 expression in non-malignant specimens (n = 34) differs up to 14.3-fold and is higher than in TNBC (p = 0.005). The Her4 expression in Her2 positive tissues is only tendentially lower than in benign tissues (p = 0.64). Figure 2B: Poorly differentiated (G3), Her2 positive tumors show lower Her4 expression levels than middle grade (G2) tumor tissues (p = 0.003). Poorly differentiated TNBC tissues (G3) have significantly lower Her4 expression levels than non-malignant tissues (p = 0.02).

### Her4 (JM-a) expression in TNBC and Her2 positive patients as a function of tumor grading

Overall the median relative Her4 (JM-a) expression level was significantly lower in TNBC (p = 0.005) but not in Her2 positive tumor tissues (p = 0.64) compared to benign breast tissues (Figure 1A). TNBC samples show lower Her4 expression levels than Her2 positive specimens (p = 0.01). Tumor samples broken down with respect to grading 2 and 3 showed that Her4 expression turned out to be expressed at lower levels in poorly differentiated (G3) tumors compared to moderately differentiated (G2) Her2 positive tumors (p = 0.003). In G3-classified TNBC specimens Her4 expression was only tendentially lower compared to G2 samples (p = 0.22) (Figure 1B).

### Her4 dependent analyses of EFS and OS of TNBC and Her2 positive patients

Her4 (JM-a) positive and negative specimens were dichotomized based on a PCR expression value < 0.6 and ≥ 0.6, respectively.

In the TNBC samples, univariable Cox regression analysis showed a significant impact of JM-a expression on OS (HR = 0.15, 95% CI [0.01; 0.70], p = 0.01) but not on EFS (HR = 0.55, 95% CI [0.16; 1.40], p = 0.22). The corresponding Kaplan-Meier survival curves are presented in Figure 2A and B. Multivariable analysis, however, shows that patient age affects the OS (HR = 1.04, 95% CI [1.01; 1.08], p = 0.017) and tumor Staging IV affects both EFS (HR = 12.40, 95% CI [2.82; 52.21], p < 0.001) and OS (HR = 8.75, 95% CI [1.61; 43.51], p = 0.007) (Table 4).

**Table 4:** Univariable and multivariable Cox-regression of Her4 (JM-a) expression (< 0.6 vs. ≥ 0.6) and clinicopathological parameters

		**Event-free survival (EFS)**		**Overall survival (OS)**	
	**Prognostic factor**	**HR (95% CI)**	**p-value**	**HR (95% CI)**	**p-value**
TNBC	JM-a univariable	0.55 (0.16; 1.40)	0.223	0.15 (0.01; 0.70)	**0.010**
	JM-a	0.66 (0.19; 2.35)	0.519	0.22 (0.01; 1.14)	0.149
	Age	1.02 (0.99; 1.05)	0.145	1.04 (1.01; 1.08)	**0.017**
	Staging				
	I	Referent	-	Referent	-
	II	0.94 (0.35; 3.00)	0.913	0.72 (0.24; 2.66)	0.585
	III	3.10 (0.93; 10.86)	0.064	3.53 (0.99; 14.00)	0.054
	IV	12.40 (2.82; 52.21)	**< 0.001**	8.75 ( 1.61; 43.51)	**0.007**
	Grading (II [ref.] vs. III)	1.30 (0.54; 3.48)	0.576	1.02 (0.41; 2.77)	0.975
Her2 pos.	JM-a univariable	0.41 (0.22; 0.76)	**0.004**	0.58 (0.29; 1.12)	0.105
	JM-a	0.50 (0.21; 1.14)	0.102	1.27 (0.45; 3.77)	0.654
	Age	1.01 (0.97; 1.04)	0.646	1.02 (0.97; 1.07)	0.392
	Staging				
	I	Referent	-	Referent	-
	II	2.74 (0.91; 11.83)	0.110	1.58 (0.40; 10.47)	0.564
	III	1.57 (0.33; 8.17)	0.567	1.47 (0.17; 12.43)	0.705
	IV	4.84 (1.18; 24.67)	**0.036**	9.80 (2.05; 71.84)	**0.008**
	Grading (II [ref.] vs. III)	0.84 (0.37; 1.92)	0.68	2.24 (0.83; 6.43)	0.115

Univariable parameters with a p-value <0.1 were included in the multivariable analysis. For the TNBC collective G1 and G2 specimens were grouped together. *HR* hazard ratio, *CI* 95% confidence interval, bold: p-values < 0.05 indicating significance.

A univariable Cox proportional hazard analysis revealed a significant, favorable impact of Her4 (JM-a) expression on EFS in Her2 positive patients (HR = 0.41, 95%-CI [0.22; 0.76], p = 0.004) but not on OS (HR = 0.58, 95%-CI [0.29; 1.12], p = 0.105). Figure 2C and D present the corresponding Kaplan–Meier survival curves of EFS and OS categorized by Her4 JM-a expression. In a multivariable model including the additional covariates age, staging and grading, only Staging IV appears to significantly affect both EFS and OS (Table 4).

### Her4 dependent analyses of EFS and OS of Her2 positive patients with respect to ER expression

**Figure 3:**
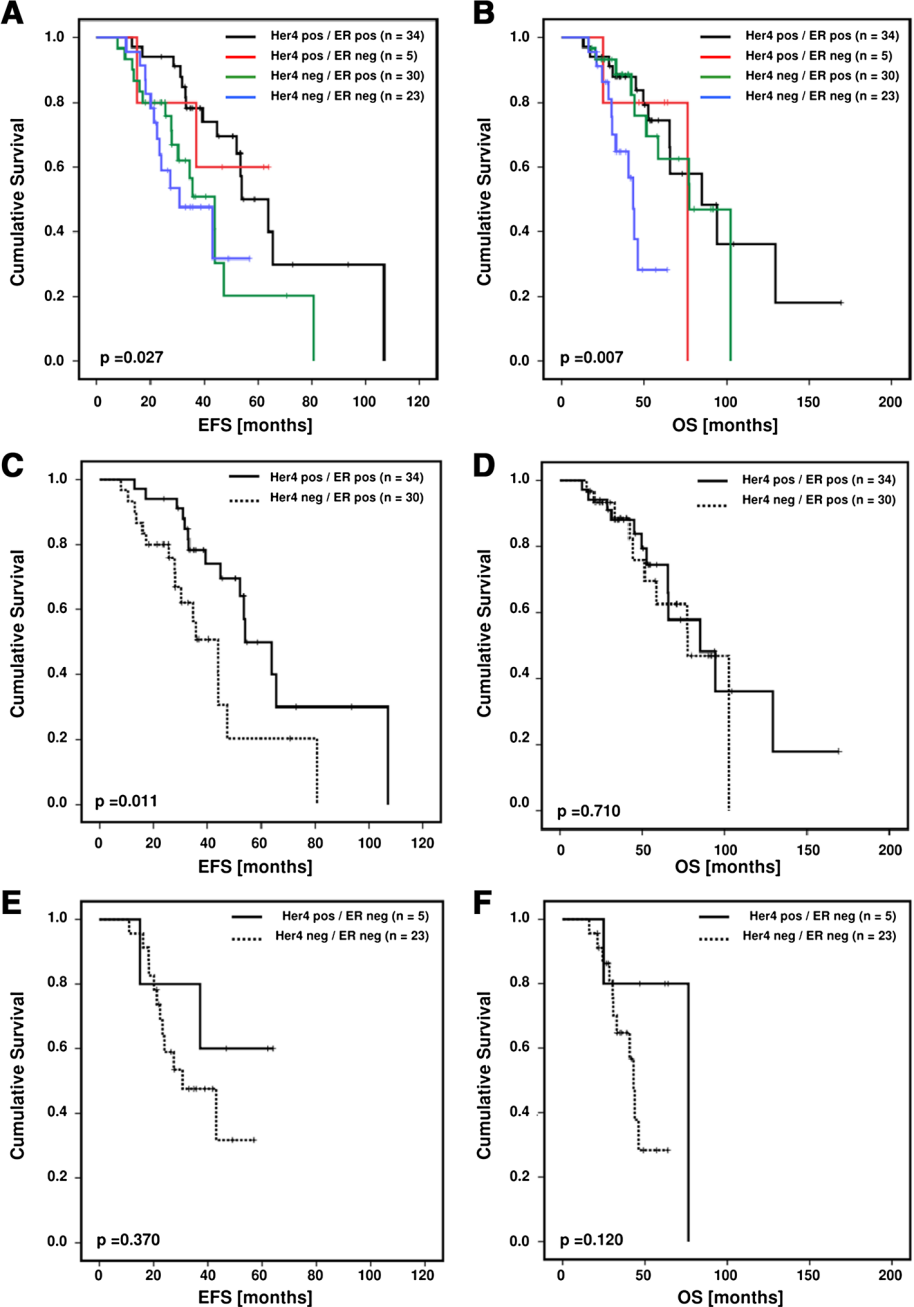
**Cumulative Kaplan-Meier curves of the effect of Her4 (JM-a) and ER expression on EFS and OS of Her2 positive patients.** Panels **A** and **B**: EFS and OS of Her2 positive Her4/ER subcollectives are shown. Panels **C – F**: Kaplan-Meier analyses of the effect of differentially classified Her4 (JM-a) and ER expression on EFS **(C and E)** and OS **(D and F)** of Her2 positive patients.

The Kaplan-Meier analysis of Her2 positive patients revealed a significant impact of Her4 expression on EFS (p = 0.027) and OS (p = 0.007) when the cohort is differentiated in terms of ER expression (Figure 3A and B). Statistically broken down to Her4/ER positive/negative cohorts (Figure 3C - E), Her4 expression turned out to be significantly associated with a prolonged EFS in Her2/ER double-positive patients (p = 0.011; Figure 3C) but not with a prolonged OS (p = 0.710; Figure 3D). No benefit from Her4 expression could be identified in Her2 positive/ER negative patients, either in terms of EFS (p = 0.370; Figure 3E) or OS (p = 0.120; Figure 3F).

### Correlation analysis (Spearman-Rho) of Her4 isoform (CYT1, CYT2) expression to clinicopathologic parameters

We analyzed the correlation (Spearman-Rho) between Her4 CYT1 and CYT2 expression and also to the clinicopathological parameters Grading and Staging (Table 5). This analysis revealed a significant positive correlation of CYT1 and CYT2 expression (r = 0.605, p < 0.001). Moreover, in Her2 positive tumors CYT1/CYT2 expression is inversely correlated with tumor grading (CYT1: r = -0.316, p = 0.002; CYT2: r = -0.298, p = 0.003), which is in agreement with the data presented in Figure 1B).

**Table 5:** Non-parametric correlations (Spearman-Rho) of Her4 receptor isoform expression (CYT1, CYT2) with clinicopathological parameters

		**CYT1**		**CYT2**		**Grading**		**Staging**	
		**r**	**p**	**r**	**p**	**r**	**p**	**r**	**p**
**TNBC**	**CYT1**	-	-	0.605	**< 0.001**	-0.206	0.076	-0.094	0.441
	**CYT2**	0.605	**< 0.001**	-	-	-0.167	0.152	-0.035	0.774
**Her2 pos.**	**CYT1**	-	-	0.595	**< 0.001**	-0.316	**0.002**	-0.220	0.051
	**CYT2**	0.595	**< 0.001**	-	-	-0.298	**0.003**	-0.033	0.776

r = correlation coefficient, p = p-value, bold: significant correlations i.e. p-value < 0.05.

## Discussion

The impact of Her4 RTK expression on the course and outcome of breast cancer disease remains largely unclear. A number of findings emerged implying a favorable effect of Her4 expression [10-13,16]. In contrast, *in-vitro* and *in-vivo* studies demonstrated inhibited tumor cell proliferation by downregulation of Her4 expression or deactivation of Her4 function upon Her4 targeting [22-24]. The retrospective study we present here reveals for the first time a favorable impact of Her4 expression on the OS of TNBC patients. In addition, we confirmed previously described indications for a beneficial impact of Her4 in Her2/ER positive patients [16]. A differential expression of Her4 isoforms does not, however, play a critical role in the course and outcome of these breast cancer subgroups.

In a multivariable Cox model with known strong predictors for OS and EFS such as age, grading and staging, Her4 expression was, however, no longer significant. This is not surprising since we were limited by the number of events in both collectives and the power to detect a significant effect of Her4 expression against other strong predictors is too low. Nevertheless we think that Her4 expression might still have a significant, independent effect on EFS and OS, which can only be demonstrated by an analysis of a larger cohort.

Accumulating data derived from preclinical investigations suggest that the apparent inconsistency regarding the importance of Her4 expression could be potentially explained by an ambivalent Her4 function i.e. pro-apoptotic [25,26] and pro-proliferative [26,27] activity. A tumor suppressive or oncogenic Her4 receptor activity might be attributed to receptor isoforms respectively expressed. Only the JM-a but not the JM-b extracellular domain is known to be ligand-independently activated by TACE-induced cleavage [18,22,27,28]. Subsequently, the intracellular domain (either CYT1- or CYT2-4ICD) can be cleaved by γ-secretase and differentially triggers downstream signaling pathways. Once released, the 4ICD differentially triggers downstream signaling pathways e.g. by translocation into the nucleus and coactivation of ER-related gene transcription, which in turn stimulates cell proliferation [2,29]. Alternatively, the Wwox protein would rather inhibit 4ICD routing into the nucleus. If not degraded by the ubiquitin ligase Itch, soluble 4ICD has been shown to interact via its BH3 subdomain with pro-apoptotic proteins (e.g. BAK) followed by increased permeability of mitochondria, cytochrom-c release, and finally cell death [15,20,25,27].

Although Her4 inherently possesses a potential bivalent activity, the expression analysis of this study suggests a favored evolvement of a tumorsuppressive activity rather than oncogenic action. This observation is supported by the finding of reduced Her4 expression in rather progressive and poorly differentiated breast tumors as revealed by our data (Figure 1B) and other studies [4,27]. Moreover, a reactivation of epigenetically silenced Her4 has been reported to induce apoptosis in breast cancer cells [30].

In Her2 positive breast cancer tissues we identified Her4 to be preferentially expressed in ER-positive rather than in ER negative specimens (Figure 3). This observation is in agreement with findings previously reported by Junttila et al. [22] and recently confirmed by Fujiwara et al. [31]. Obviously, the Her4 receptor develops its favorable impact primarily in the presence of ER, which in turn suggests a functional Her4 (4ICD)/ER interaction. This consideration is supported by the observation that the favorable impact of Her4 expression loses its significance in the Her2 positive/ER negative collective, both in terms of EFS (p = 0.370) and OS (p = 0.120). In contrast, the outcome (OS) of TNBC patients, who are typically ER negative, is significantly better when the tumor specimens appear Her4 positive (p = 0.030). Taking these findings together, the evolvement of a favorable (tumor suppressive) impact of Her4 expression in Her2/ER double-positive tumor patients is apparently inconsistent with a pro-proliferative activity that has been described *in-vitro*. Moreover, the Her4 receptor seems to restrain tumor growth even in the absence of ER expression, as shown for the TNBC collective. Within the period of observation, only 2 out of 12 Her4 positive TNBC patients suffered from a local recurrence. Accordingly, the favorable impact of Her4 expression is more pronounced in terms of OS (p = 0.03) than in terms of EFS (p = 0.257).

With respect to differential Her4 isoform expression, a preferred expression of CYT1 over CYT2 (or *vice versa*) intracellular domain, or a pronounced effect of high or low CYT1/CYT2 expression ratios cannot be concluded either from our data or other studies [22]. One might speculate that the functional diversity that has been attributed to the intracellular domain by pre-clinical studies [3,32,33], can either not be deduced by a descriptive study or does not, in fact, play a relevant role *in-vivo*. Instead, the identification of Her4 either by immunohistochemistry [10-13], fluorescence *in-situ* hybridization (FISH) [14,16], or qPCR [22] seems to be sufficient for attributing a positive impact on the course/outcome of breast cancer disease. Since JM-b isoforms are never expressed and CYT1/CYT2 intracellular domains are always simultaneously expressed, a diagnostic differentiation of Her4 isoforms is obviously not informative.

Considering a more translational approach, it could be evaluated to what extent the Her4 receptor represents a potential target that could be therapeutically utilized in 18% of TNBC and in 43% of Her2 positive breast cancers. As with ER, which basically represents a favorable prognostic marker as well, this hormone receptor is being very successfully targeted with e.g. tamoxifen or equivalent chemicals. Preclinical studies have revealed that anti-Her4 targeting with a newly developed antibody Ab1479 attenuates receptor activity and in turn reduces the formation of proliferative cell colonies [18,24,34]. Hence, even if the presence of a given biomarker (ER, Her4) is strongly correlated with a favorable outcome of disease, targeting this biomarker might be a potential beneficial therapeutic strategy.

## Conclusion

Overall, our study reveals a positive impact of Her4 (JM-a) expression in triple-negative (OS) and Her2/ER-positive (EFS) breast cancer. The ever-growing body of evidence supporting the favorable impact of Her4 expression in breast cancer suggests the need to reexamine the commonly accepted idea that (over-)expression of (receptor) tyrosine kinases is necessarily associated with oncogenic activity. Only further extensive functional *in-vitro* and *in-vivo* analyses focusing on the importance of Her4 in the context of differential Her receptor co-expression will facilitate the consideration of this important receptor in individually optimized therapy based on a modular approach [35].

## Abbreviations

4ICD: Her4 receptor intracellular domain; cDNA: Complementary deoxyribonucleic acid; CYT1: Cytoplasmatic splice variant-1; CYT2: Cytoplasmatic splice variant-2; dNTP: Deoxynucleotide triphosphate; EFS: Event-free survival; e. g.: *Exempli gratia* (for example); ER: Estrogen receptor; Her: Human epidermal growth factor related receptor; i. e.: *Id est* (that is); JM-a: Juxtamembrane splice variant a; JM-b: Juxtamembrane splice variant b; OFS: Overall survival; qPCR: Quantitative polymerase chain reaction; RNA: Ribonucleic acid; TACE: Tumor-necrosis-factor-α-converting enzyme; TNBC: Triple-negative breast cancer; RTK: Receptor tyrosine kinase; Wwox: WW domain-containing oxidoreductase.

## Competing interests

The authors declare no competing interests.

## Authors’ contributions

AM performed the major part of the experimental work. SB contributed to the study draft and data interpretation. SDD contributed to the manuscript draft. FZ performed advanced statistical analysis and data interpretation. OO contributed to the study draft and data interpretation. GB designed the study and wrote the manuscript. All authors read and approved the final manuscript.

## Pre-publication history

The pre-publication history for this paper can be accessed here:

http://www.biomedcentral.com/1471-2407/13/437/prepub
